# Castleman’s Disease With Tongue Lesions in an HIV-Positive Patient From Saudi Arabia

**DOI:** 10.7759/cureus.50177

**Published:** 2023-12-08

**Authors:** Ali Alsaeed, Abdullah AlKhalaf, Abdulrahman Alhudaifi, Albatool A Alwesaibi, Ali Al Muhaif, Waad Alzaher, Sana Alsolami

**Affiliations:** 1 Internal Medicine, Dammam Medical Complex, Dammam, SAU; 2 Anatomical Pathology, Dammam Medical Complex, Dammam, SAU

**Keywords:** pneumocystis jirovecii pneumonia, lymphoproliferative disorder, human herpesvirus 8 (hhv8) infection, hiv, castleman's disease

## Abstract

Castleman's disease (CD) is a rare lymphoproliferative disorder that presents with heterogeneous clinical manifestations. We present a case report of a 34-year-old male patient co-infected with CD and HIV, who exhibited tongue lesions along with systemic symptoms. Prompt diagnosis and appropriate management, including antiretroviral therapy (ART) and targeted treatment for CD, resulted in significant clinical improvement. This case highlights the challenges faced in managing CD with tongue involvement in the setting of HIV infection and emphasizes the importance of comprehensive management strategies.

## Introduction

Human herpesvirus 8 (HHV-8) belongs to the herpesvirus family (Herpesviridae) and has a propensity to infect various cell types, including endothelial cells, dendritic cells, and lymphocytes. It is closely associated with the development of Kaposi’s sarcoma, primary effusion lymphoma, and Castleman’s disease (CD).

The prevalence of HHV-8 among Saudi Arabian patients, particularly those with HIV, remains largely unknown, with only a few reported cases. In the Middle East, HHV-8 seroprevalence has been adequately assessed in only a handful of populations, such as the Ashkenazi and Sephardic groups in Israel [1].

CD, named after Dr. Castleman, a pathologist at Massachusetts General Hospital, was initially described as a rare Lymphoproliferative disorder [2]. It is characterized by lymph node enlargement and can be categorized into two major subtypes: Unicentric CD (UCD) and Multicentric CD (MCD).

Dr. Castleman’s initial description involved a patient who experienced prolonged fever and weakness over several years and was eventually diagnosed with a large mediastinal mass through fluoroscopy. Further medical evaluation revealed mediastinal lymphadenopathy, and surgical excision unveiled the strikingly abnormal architecture of the affected lymph nodes that has since become characteristic of CD.

On the other hand, tongue involvement in CD is an infrequent manifestation that presents both diagnostic and therapeutic challenges. Here, we present a case of CD with tongue lesions and severe systemic symptoms in an HIV-positive patient, shedding light on the clinical implications and management considerations [3].

## Case presentation

We present a 34-year-old Saudi male patient who was previously healthy. He presented to the Infectious Disease Clinic at Dammam Medical Complex, a well-known HIV center in the kingdom, with a four-week history of dysphagia, significant weight loss (10 kg), and recurrent fever over the past three months. Upon his presentation, it was surprising to us that he was using a wheelchair to enter the clinic, which he attributed to tiredness and fatigue.

**Figure 1:**
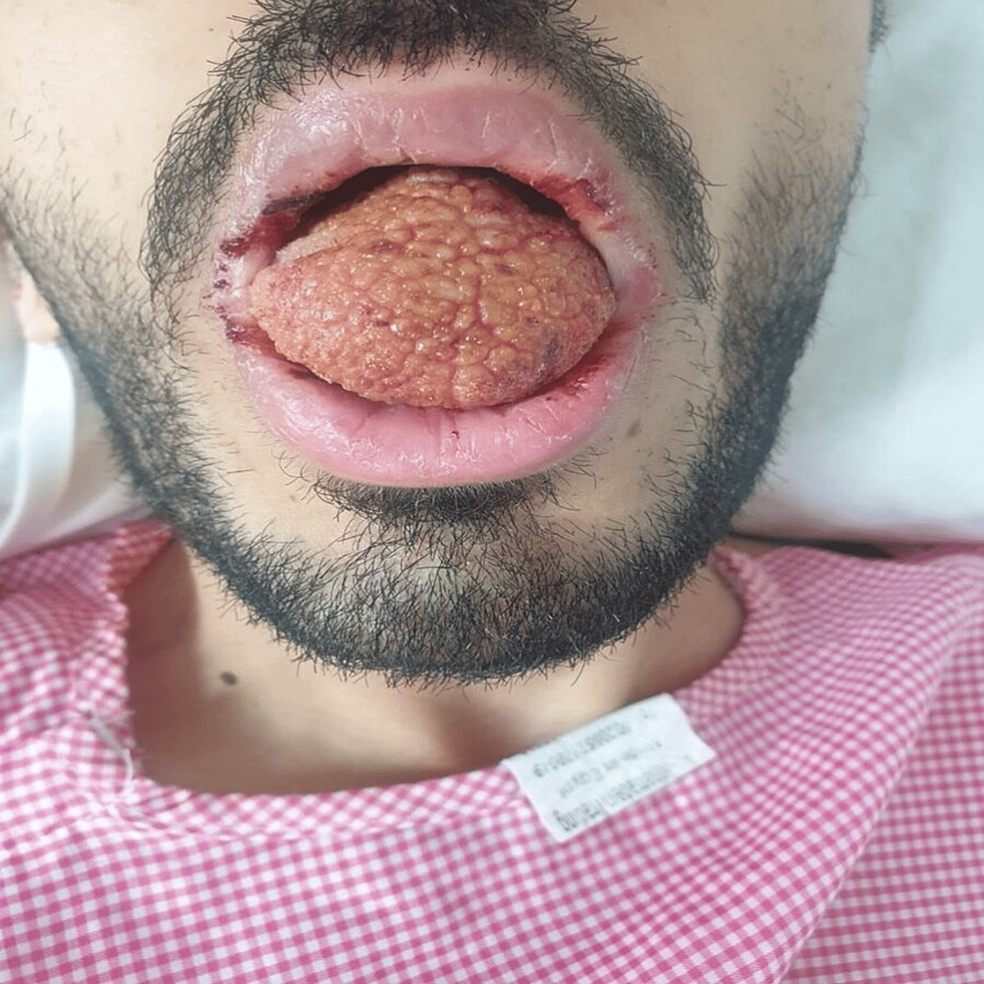
Tongue lesions at the time of presentation

Further clinical examination revealed significant tongue lesions (Figure 1). The tongue lesions observed in the patient presented with several distinct characteristics. They appeared as raised, erythematous (red) areas on the dorsum of the tongue, extending towards the lateral borders. The lesions exhibited a granular or nodular texture upon visual examination. The affected areas of the tongue were tender and painful, causing significant discomfort to the patient, especially during feeding and swallowing.

The size of the lesions varied, ranging from small discrete patches to larger confluent areas, involving multiple regions of the tongue. Some lesions exhibited irregular borders, while others had a more defined outline. The color intensity of the lesions varied from mild to moderate erythema, indicating inflammation and increased vascularity. Additionally, there were no signs of ulceration or discharge observed on the tongue.

At the time of his clinic visit, he was experiencing active respiratory symptoms, including shortness of breath and a productive cough. Given the concerning symptoms, the patient was immediately admitted to the hospital for further evaluation.

During the diagnostic workup, HIV testing confirmed a positive result the next day. Additionally, the patient was diagnosed with Pneumocystis jirovecii pneumonia, which required treatment with Bactrim and steroids.

Further evaluation revealed generalized lymphadenopathy and massive splenomegaly through physical examination and a pan-CT scan. These findings raised concerns about the possibility of lymphoma or CD.

As the patient’s platelet count was above 50,000, the surgical team was consulted for a biopsy of both his tongue and lymph node. The procedure was obtained uneventfully.

Diagnostic workup

A biopsy of the axillary nodes and tongue was performed, confirming the diagnosis of CD. Concurrently, the patient initiated antiretroviral therapy (ART) with Bictegravir/emtricitabine/tenofovir alafenamide.

**Figure 2:**
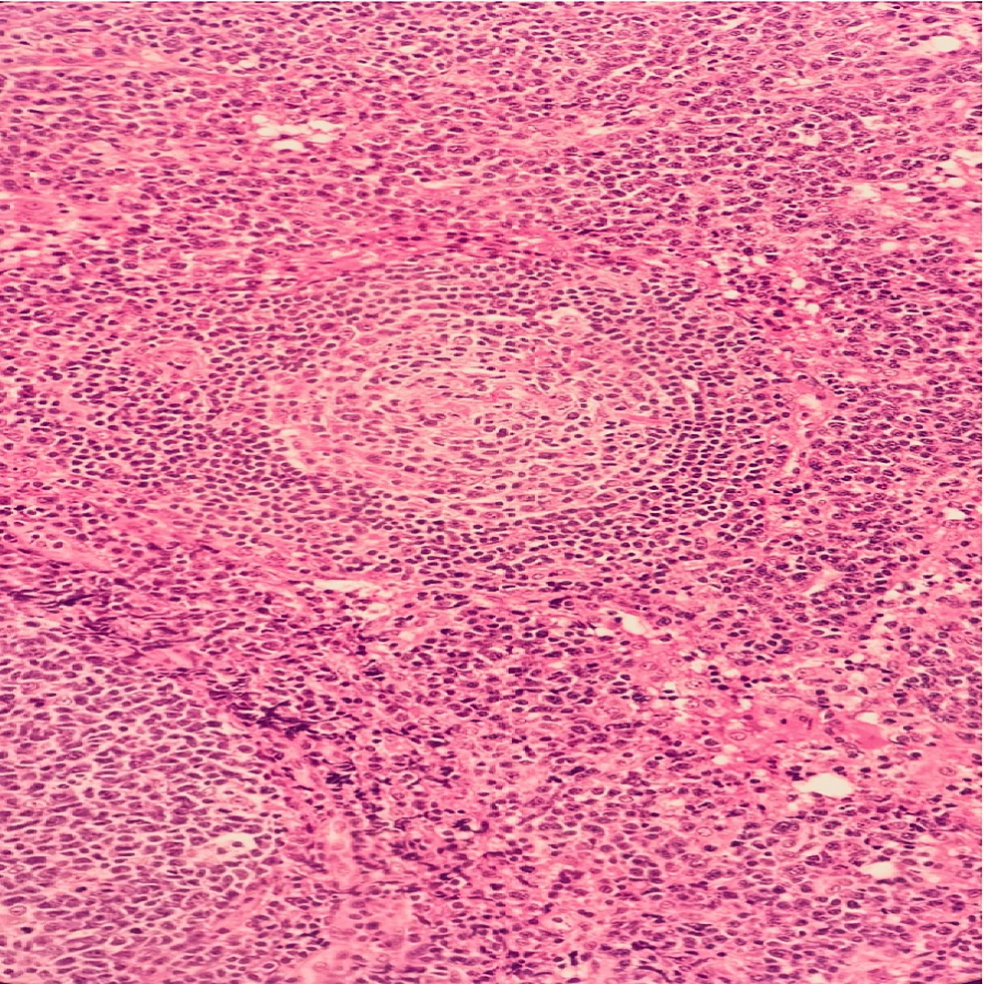
H&E, 10x: Variable reactive lymphoid follicular hypeprlasia

**Figure 3:**
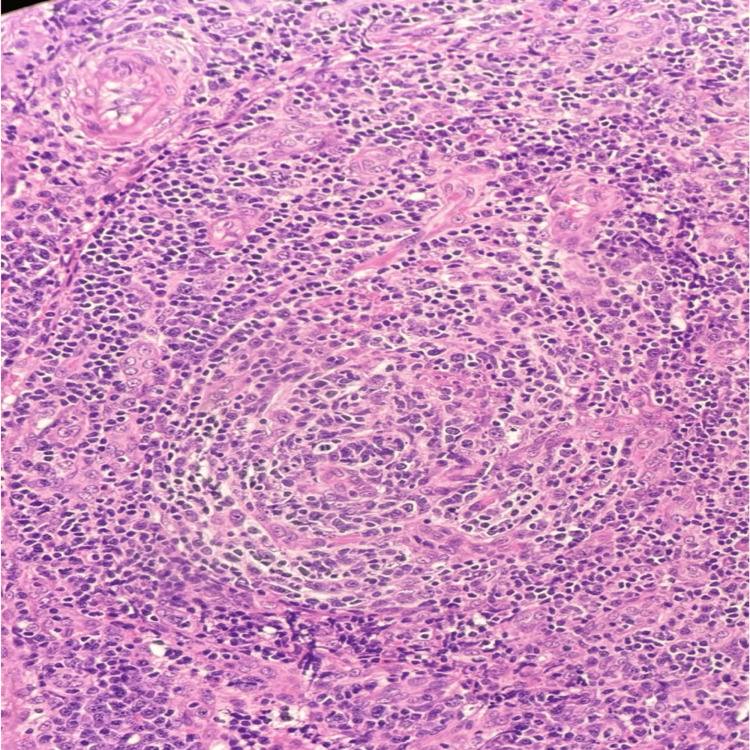
H&E, 40x: Vascular proliferation in the interfollicular area along with mixed small lymphocytes, histiocytes and plasma cells.

**Figure 4:**
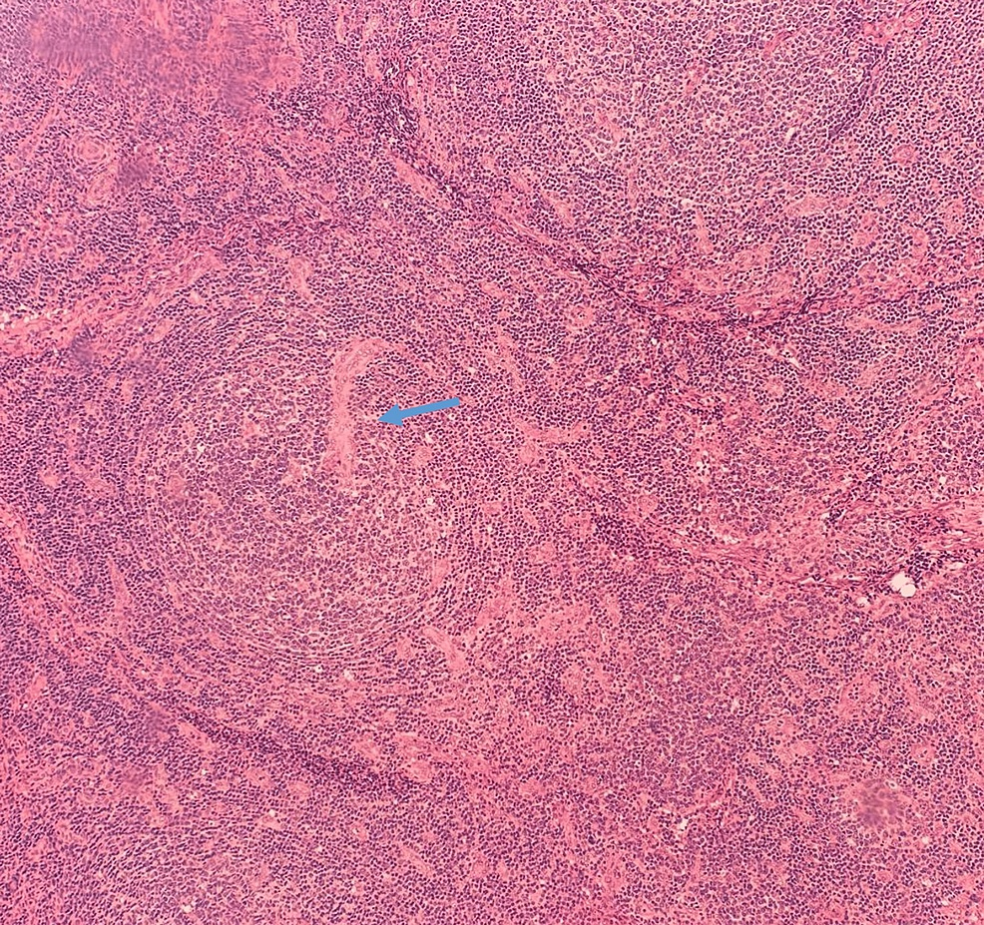
H&E, 20x: Variably sized hyperplastic follicles, surrounded by thickened mantle zones and sheets of plasma cells. A germinal center is traversed by a penetrating vessel “Lollipop Follicle” (arrow).

Laboratory investigations revealed a CD4 count of 90 cells/μL and an HIV viral load of 1,000,000 copies/mL, confirming the patient’s AIDS status. Pancytopenia and severe anemia were also observed, necessitating blood transfusions during the admission course. The biopsy results (Figures 2-4) demonstrated reactive lymphocytes, HHV8 infection, interfollicular plasma cells, and other immunostaining patterns consistent with CD [4].

Treatment and clinical course

The patient’s respiratory symptoms worsened, necessitating high-flow nasal cannula support and transfer to the intensive care unit (ICU) for monitoring. Oral feeding became challenging, leading to the need for nasogastric tube feeding. Importantly, the patient did not require intubation or inotropic support during their ICU stay.

Managing CD with tongue involvement in an HIV-positive patient necessitates a multidisciplinary approach. The treatment goals include controlling the underlying HIV infection, managing CD, and addressing associated complications.

The clinical course of MCD is characterized by recurrent attacks with systemic symptoms, lymphadenopathy, splenomegaly, cytopenia, and inflammation associated with a high HHV-8 viral load in peripheral blood mononuclear cells [5,6].

Antiretroviral therapy

Initiating effective ART is crucial to suppress the HIV viral load, restore immune function, and reduce the risk of opportunistic infections. In this case, the patient began ART with Bictegravir/emtricitabine/tenofovir alafenamide, a combination of bictegravir, emtricitabine, and tenofovir alafenamide. The ART regimen was tailored based on the patient’s HIV resistance profile and comorbidities.

CD-specific treatment

Treatment for CD varies depending on the subtype and disease extent. In this case, the patient was diagnosed with MCD associated with human herpesvirus 8 (HHV8) infection. Therapeutic options for MCD include the following.

Anti-Interleukin-6 (IL-6) Therapy

IL-6 plays a crucial role in the pathogenesis of CD. Monoclonal antibodies targeting IL-6, such as tocilizumab, have shown efficacy in controlling disease activity and improving symptoms.

Chemotherapy

In some cases, chemotherapy may be necessary, especially in aggressive or refractory forms of CD. Therapeutic agents, such as rituximab, cyclophosphamide, and corticosteroids, may be used to induce remission and reduce disease burden [4].

Several antiviral medications have shown the ability to effectively inhibit HHV-8 replication in vitro, including ganciclovir, foscarnet, and cidofovir [7,8]. A limited number of patients with HHV-8-associated MCD have been treated with antiviral medications with varying success. Among four patients treated with foscarnet, two showed no improvement [9], while two experienced persistent remission for 12-24 months [10].

Various factors may contribute to the heterogeneous response to antiviral therapy for HHV-8-associated MCD. First, because current in vitro models may not adequately predict in vivo efficacy, clinical studies are needed to determine which antiviral medications have the most significant impact on HHV-8. Additionally, the optimal timing for administering antivirals remains unclear [11].

The patient discussed the situation with the medical team and was presented with treatment options. A shared decision between the Infectious Disease team and the patient was to initiate an ART trial before considering potentially toxic medications, particularly given the patient’s condition at that time.

Supportive care measures are essential for managing complications and improving patient outcomes. In this instance, the patient necessitated blood transfusions to address severe anemia and pancytopenia. Additionally, providing nutritional support, closely monitoring vital signs, and effectively managing concurrent infections, such as Pneumocystis jirovecii pneumonia, were integral components of the supportive care plan.

Clinical course

**Figure 5:**
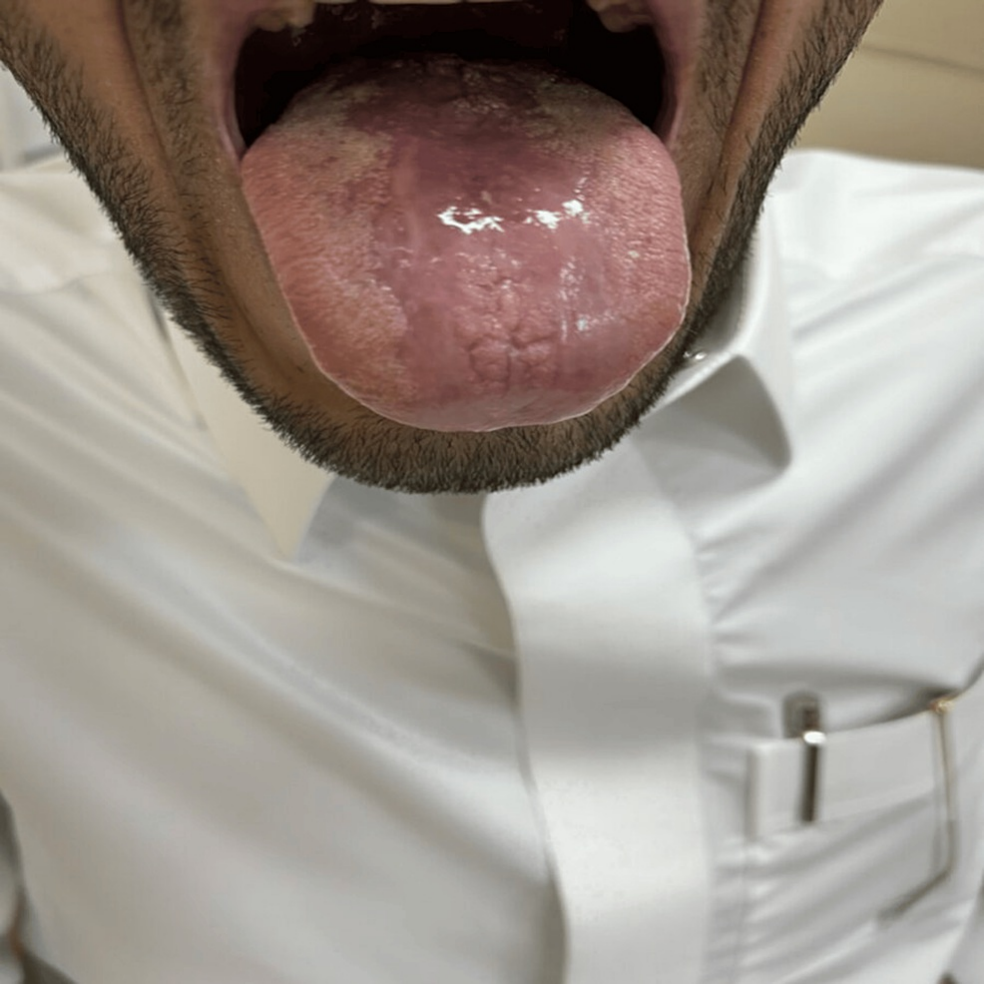
After four weeks from starting ART.

The patient’s respiratory symptoms worsened during the initial phase of treatment, necessitating admission to the ICU and high-flow nasal cannula support. Nevertheless, following the initiation of ART and the implementation of supportive care measures, the patient’s condition progressively ameliorated. After a two-week period, the patient’s condition stabilized sufficiently to permit discharge. Subsequent follow-up examinations revealed a significant improvement in tongue lesions (Figure 5) and favorable changes in complete blood count parameters.

## Discussion

Currently, there is limited data available that focuses on the prevalence of CD in specific HIV-positive populations, CD is a rare and heterogeneous disorder that can occur in both HIV-positive and HIV-negative individuals [12]. The incidence of UCD has been estimated at 16 to 19 cases per million in the US population, which translates to 5,000 to 6,000 patients per year [13]. However, there is still no clear data available about the incidence of UCD in Saudi Arabia.

CD involving the tongue is a rare manifestation that can complicate the management of HIV-positive patients. In this case, tongue lesions were a prominent feature, and the patient experienced severe systemic symptoms requiring ICU care. The timely initiation of ART and targeted therapy for CD resulted in significant clinical improvement.

Our study focused on the therapeutic challenges associated with CD, particularly in the context of HIV-positive individuals. The case report highlighted the significance of early recognition of tongue involvement and the implementation of comprehensive management strategies, including ART and specific CD treatments, to achieve optimal outcomes. These findings are consistent with the therapeutic complexities observed in previous studies [1].

Our study contributes to the growing body of literature on CD, particularly within the context of HIV co-infection. The need for further research into tongue involvement in CD, especially in the presence of HIV, is underscored by the unique challenges and complexities observed in our patient cohort. This aligns with the call for continued investigation and understanding of CD, as emphasized by leading experts in the field.

The multidisciplinary approach employed in this study, involving experienced healthcare professionals and researchers, reflects the importance of collaborative efforts in addressing the complexities of CD, especially in the presence of comorbid conditions such as HIV. This aligns with the recommendations for a multidisciplinary approach advocated by experts in the field.

In conclusion, our study sheds light on the therapeutic challenges and complexities associated with CD, particularly in the context of HIV co-infection. The findings underscore the importance of early recognition, comprehensive management strategies, and the need for further research to advance the understanding and management of CD, especially in the presence of tongue lesions in HIV-positive individuals. This example demonstrates how the discussion section can effectively integrate the study's findings with existing literature, ethical considerations, and the need for further research in the field of CD.

## Conclusions

HIV itself may predispose individuals to the development of CD, and symptoms can be severe, with flares of the disease possible at any CD4 count. HIV-associated MCD generally exhibits a rapidly progressive clinical course.

The presence of tongue lesions in CD presents diagnostic and therapeutic challenges, especially in HIV-positive individuals. Early recognition of tongue involvement and the implementation of comprehensive management strategies, including ART and specific CD treatments, is crucial for achieving optimal outcomes. This case underscores the importance of an experienced multidisciplinary approach and emphasizes the need for further research into tongue involvement in CD, particularly within the context of HIV co-infection.

## References

[1] Jalilvand S, Shoja Z, Mokhtari-Azad T, Nategh R, Gharehbaghian A (2011). Seroprevalence of human herpesvirus 8 (HHV-8) and incidence of Kaposi's sarcoma in Iran. Infect Agent Cancer.

[2] Castleman B, Towne VW (1954). Case records of the Massachusetts General Hospital: Case No. 40231. N Engl J Med.

[3] Carbone A, Borok M, Damania B (2021). Castleman disease. Nat Rev Dis Primers.

[4] Abramson JS (2019). Diagnosis and management of Castleman disease. J Natl Compr Canc Netw.

[5] Oksenhendler E, Duarte M, Soulier J (1996). Multicentric Castleman's disease in HIV infection: a clinical and pathological study of 20 patients. AIDS.

[6] Du MQ, Bacon CM, Isaacson PG (2007). Kaposi sarcoma-associated herpesvirus/human herpesvirus 8 and lymphoproliferative disorders. J Clin Pathol.

[7] Kedes DH, Ganem D (1997). Sensitivity of Kaposi's sarcoma-associated herpesvirus replication to antiviral drugs. Implications for potential therapy. J Clin Invest.

[8] Neyts J, De Clercq E (1997). Antiviral drug susceptibility of human herpesvirus 8. Antimicrob Agents Chemother.

[9] Bottieau E, Colebunders R, Schroyens W (2000). Multicentric Castleman's disease in 2 patients with HIV infection, unresponsive to antiviral therapy. Acta Clin Belg.

[10] Nord JA, Karter D (2003). Low dose interferon-alpha therapy for HIV-associated multicentric Castleman's disease. Int J STD AIDS.

[11] Casper C (2008). New approaches to the treatment of human herpesvirus 8-associated disease. Rev Med Virol.

[12] Fajgenbaum Fajgenbaum, D. C., Uldrick Uldrick (2017). International, evidence-based consensus diagnostic criteria for HHV-8-negative/idiopathic multicentric Castleman disease. https://ashpublications.org/blood/article/129/12/1646/36100/International-evidence-based-consensus-diagnostic.

[13] van Rhee F, Oksenhendler E, Srkalovic G (2020). International evidence-based consensus diagnostic and treatment guidelines for unicentric Castleman disease. Blood Adv.

